# Dual Regulation of Myocardin Expression by Tumor Necrosis Factor-α in Vascular Smooth Muscle Cells

**DOI:** 10.1371/journal.pone.0112120

**Published:** 2014-11-10

**Authors:** Pavneet Singh, Xi-Long Zheng

**Affiliations:** Smooth Muscle Research Group, Libin Cardiovascular Institute of Alberta, Department of Biochemistry & Molecular Biology, Cumming School of Medicine, University of Calgary, Calgary, Alberta, Canada; University of Iowa, United States of America

## Abstract

De-differentiation of vascular smooth muscle cells (VSMCs) plays a critical role in the development of atherosclerosis, a chronic inflammatory disease involving various cytokines such as tumor necrosis factor-α (TNFα). Myocardin is a co-factor of serum response factor (SRF) and is considered to be the master regulator of VSMC differentiation. It binds to SRF and regulates the expression of contractile proteins in VSMCs. Myocardin is also known to inhibit VSMC proliferation by inhibiting the NF-κB pathway, whereas TNFα is known to activate the NF-κB pathway in VSMCs. NF-κB activation has also been shown to inhibit myocardin expression and smooth muscle contractile marker genes. However, it is not definitively known whether TNFα regulates the expression and activity of myocardin in VSMCs. The current study aimed to investigate the role of TNFα in regulating myocardin and VSMC function. Our studies showed that TNFα down-regulated myocardin expression and activity in cultured VSMCs by activating the NF-κB pathway, resulting in decreased VSMC contractility and increased VSMC proliferation. Surprisingly, we also found that TNFα prevented myocardin mRNA degradation, and resulted in a further significant increase in myocardin expression and activity in differentiated VSMCs. Both the NF-κB and p44/42 MAPK pathways were involved in TNFα regulation of myocardin, which further increased the contractility of VSMCs. These differential effects of TNFα on myocardin seemingly depended on whether VSMCs were in a differentiated or de-differentiated state. Taken together, our results demonstrate that TNFα differentially regulates myocardin expression and activity, which may play a key role in regulating VSMC functions.

## Introduction

Atherosclerosis is generally classified as a chronic inflammatory disease in which cytokines play an important role. It involves lipid accumulation in the vessel wall, inflammation, macrophage and vascular smooth muscle cell (VSMC) apoptosis, proliferation and migration. The abnormal proliferation of VSMCs plays an important role in the pathogenesis of atherosclerosis and hypertension [1]. Cytokines regulate VSMC functions and are considered important for the pathogenesis of vascular diseases [2], [3]. Tumor necrosis factor-α (TNFα) is a pleiotropic cytokine produced by a variety of cells including macrophages, endothelial cells, and SMCs. It is associated with the progression of atherosclerotic disease by inducing the production of interleukin 6 and chemokines, the adherence of leukocytes to the endothelium, as well as affecting lipid metabolism [4], [5].

In the normal state, the blood vessel contains VSMCs which are in a differentiated, contractile and non-proliferative quiescent state. However, when there is vascular injury or during the development of atherosclerosis, VSMCs de-differentiate and shift to a synthetic phenotype so as to actively proliferate and migrate, leading to the development of lesions [6].

Expression of the majority of VSMC contractile genes is dependent on the presence of CC(A/T)_6_GG (CArG) boxe(s) in their promoters/enhancers. Serum response factor (SRF) is essential for the regulation of muscle-specific genes through its interaction with the muscle-enriched SRF cofactor myocardin [7]. Myocardin is a muscle-specific co-transcriptional factor of the SAP domain family of transcription factors and is predominantly expressed in cardiomyocytes and SMCs. Myocardin stimulates SRF activity by forming a ternary complex with SRF on DNA and presents its strong transcriptional activation domain to SRF, which otherwise is a very weak activator of transcription. Myocardin is the master regulator of SMC differentiation and is responsible for phenotypic modulation of VSMCs by regulating the expression of contractile proteins, such as ACTA2, ACTG2, MYH11, MYLK, TAGLN, CNN1 and caldesmon [8], [9], [10].

Myocardin has also been shown to inhibit cellular proliferation by inhibiting NF-κB (p65)-dependent cell cycle progression in SMCs [11]. The phenotypic modulation of VSMCs from a contractile to synthetic state plays an important role in the development of atherosclerosis, which is modulated in part by inflammatory cytokines like TNFα [6]. TNFα is well known to activate the NF-κB pathway in inflammation [12], resulting in a multitude of effects on VSMCs. Blocking the NF-κB pathway with the NF-κB inhibitory peptide, IκBa, leads to prevention of VSMC proliferation [12]. Moreover, NF-κB activation decreases the expression levels of myocardin and smooth muscle differentiation marker genes [13]. TNFα has also been shown to inhibit smooth muscle contractile marker genes in cultured rat cerebral VSMCs [14]. Taken together, these findings suggest that TNFα may be involved in the regulation of myocardin expression and activity in VSMCs. However, there has not been much known about the regulation of myocardin by cytokines. Our current study revealed that TNFα regulates VSMC function by differentially regulating myocardin expression and activity.

## Materials and Methods

### Reagents and antibodies

Dulbecco's Modified Eagle Medium (DMEM), fetal bovine serum (FBS) and rat-tail collagen-I were purchased from Invitrogen (Burlington, Canada). TNFα, propidium iodide (PI), doxycycline (Dox) and antibodies for CNN1 and β-actin were purchased from Sigma-Aldrich (Oakville, ON, Canada). Antibodies for myocardin (M-16), TAGLN and p65 were purchased from Santa Cruz (Santa Cruz, CA). p44/42 MAPK and phospho-p44/42 MAPK antibodies were purchased from Cell Signaling Technology (Beverly, MA, USA).

### Cell culture, tetracycline-inducible expression system, transient transfection

Human aortic SMCs (CRL-1999) were purchased from ATCC (Manassas, VA). A tetracycline-inducible expression system (T-REx, Invitrogen, Canada) for wild-type Flag-tagged mouse myocardin (a kind gift from Dr. Da-Zhi Wang, Harvard Medical School Children Hospital) or empty vector (TR) in human aortic SMCs was established as previously described [11]. Cells were grown in DMEM/F-12 Ham supplemented with 10% tetracycline-reduced FBS, penicillin (100 units/ml), and streptomycin (100 µg/ml) at 37°C in a humidified atmosphere of 95% air and 5% CO_2_. VSMCs were cultured for 24 h and were then serum-starved for 24 h before treatment with TNFα (50 µg/ml). Expression of myocardin was induced by 1 µg/ml Dox.

### Western blot analysis

Cells were washed with PBS 3 times and were then lysed for protein extraction using non-reducing sample buffer (50 mM Tris–HCl, pH 6.8, 2% (w/v) sodium dodecyl sulfate (SDS), 10% (v/v) glycerol), supplemented with protease inhibitor cocktail and phenylmethanesulfonyl fluoride. Equal amounts of protein from each sample were separated by SDS-PAGE as described before [11]. Proteins were then transferred to nitrocellulose membrane and blocked with 5% skim-milk. Membranes were then incubated in specific primary antibodies and horseradish-peroxidase-coupled secondary antibodies. The protein bands were visualized using chemiluminesence (ECL reagent, GE Healthcare).

### Reverse transcription-qPCR

Total RNA was extracted from cultured cells using a RNA isolation kit (Qiagen,Mississauga, Canada) and quantified with a NanoDrop spectrophotometer. 1 µg of total RNA was used for cDNA synthesis using the iScript cDNA Synthesis Kit (Bio-Rad, Mississauga, Canada). qPCR was performed using pre-designed primers (Qiagen). The gene expression was normalized using the glyceraldehyde 3-phosphate dehydrogenase (GAPDH), a housekeeping gene, as described before [15]. Since over-expressed myocardin is of mouse origin, we used human Hs_Myocd primers to detect endogenous myocardin expression and mouse Mm_myocardin primers to detect the over-expressed myocardin.

### Collagen gel lattice contraction assay

An *in vitro* collagen contraction assay was performed to determine the contractility of VSMCs as described before [16]. VSMCs were serum-starved for 24 h and were then trypsinized, centrifuged, and counted with hemocytometer. 1.52×10^5^ cells/ml/well of VSMCs were mixed with a collagen solution as previously described [16], followed by seeding into a 24-well plate. The plate was then kept in an incubator at 37°C and 5% CO_2_ for 30 min for the gel to solidify. 500 µl of DMEM/F-12 Ham medium was added to each well after the gel was formed. The gels were then detached with a 200 µl pipette tip and appropriate treatment was added. For each treatment, the gels were photographed using a Nikon Coolpix 995 digital camera, the contraction was calculated by determining gel areas in Image J software and the data were expressed as percentage of the area of the control.

### NF-κB activation assay

Two methods were used to determine NF-κB activation. The first method was modified from the previous protocol [17], which used Laser Scanning Cytometry (LSC) to detect NF-κB activation. Briefly, after culturing VSMCs for 24 h on coverslips, we serum-starved the cells and then treated them with TNFα for different time periods. The cells were then fixed using 80% ethanol, followed by treatment with 0.1% Triton-X 100. The cells were then blocked with 2% skim-milk for 30 min before being incubated with p65 antibody for 60 min. The cells were then washed with 1X PBS before being incubated with Alexa Fluor (AF) 488-conjugated goat secondary antibody for 60 min. The cells were then washed with 1X PBS and the nuclei were counter-stained with PI. The cover-slips were mounted and scanned using LSC. NF-κB activation was determined by detecting the p65 subunit in the nuclei of VSMCs, resulting from its dissociation and subsequent nuclear translocation. To quantify the p65 nuclear translocation, we scanned the nuclei of the cells and detected p65 in the nuclei by detecting the AF488 signals. The results were obtained as an integral of AF488 signal (Y-axis) and PI signal (X-axis). An increase in AF488 signal on Y-axis indicated an increase in nuclear translocation of p65. The results were presented as the % of p65 positive nuclei versus the total number of nuclei.

The second method we used to determine NF-κB activation was western blot detection of the nuclear translocation of the p65 subunit as described previously [18]. VSMCs were serum-starved for 24 h and then treated with TNFα for different time periods. The nuclear and the cytoplasmic fractions were isolated, followed by determination of protein concentration using the Bio-Rad Protein Assay (Bio-Rad, UK). Equal amounts of protein were loaded in each well of the SDS-PAGE gel and western blot analysis was performed as described above to detect the p65 subunit in both the nuclear and cytoplasmic fractions. NF-κB activation was indicated by an increase in nuclear localization of the p65 subunit.

### Bromodeoxyuridine (BrdU) incorporation assay for VSMC proliferation

BrdU incorporation assays have been extensively used to determine cell proliferation, because proliferating cells actively incorporate BrdU into their DNA. VSMCs were cultured on coverslips in DMEM/F-12 Ham with 10% FBS for 24 h and were then serum-starved for 24 h. Thereafter, appropriate treatments were done for 24 h before the cells were labeled with BrdU for 60 min. The cells were fixed with 80% ethanol and permeabilized with 0.1% Triton-X 100. To assay DNA synthesis, cells were stained with BrdU monoclonal antibody and the nuclei were counter-stained with PI. After staining, BrdU-positive cells were analyzed by LSC as described previously [11].

### Statistical analysis

Data are expressed as mean ± S.E.M. The number of replicates (n) represents the number of independent assays. Differences were evaluated using the Student's t-test or one-way ANOVA with Instat3.0 (Graphpad Software, La Jolla, CA).

## Results

### TNFα down-regulates myocardin expression and activity in cultured VSMCs

**Figure 1:**
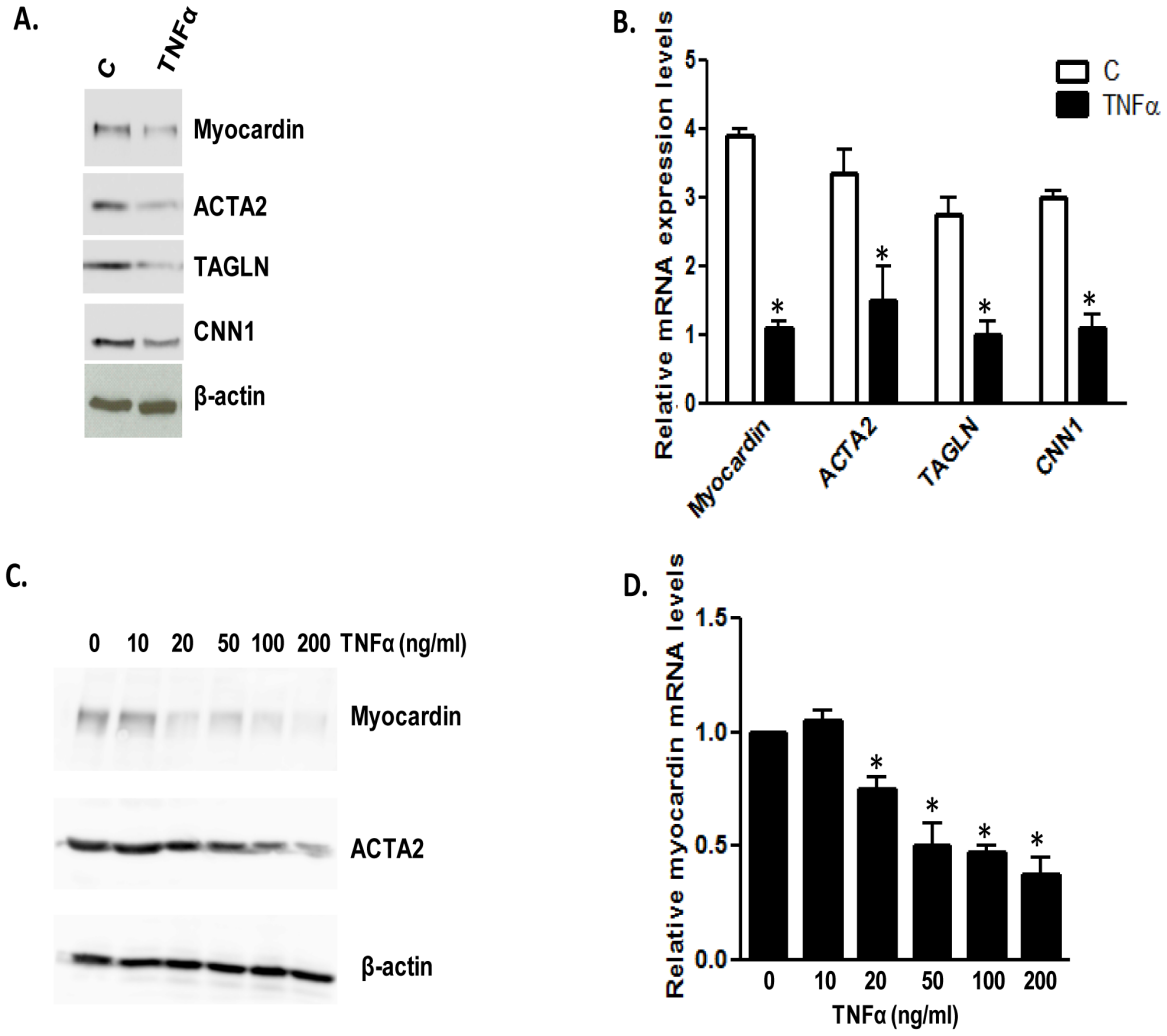
TNFα down-regulates endogenous myocardin expression and activity. VSMCs were serum-starved for 24 h and treated with TNFα (50 ng/ml) for 24 h. (**A**) Western blot analysis showing the effects of TNFα on myocardin and contractile marker protein expression levels compared with the control (C). (**B**) RT-qPCR analysis showing the effect of TNFα on myocardin and contractile marker mRNA expression levels compared to control (C). (**C,D**) Western blot and RT-qPCR analysis showing the concentration dependent effects of TNFα on myocardin expression and activity. qPCR results were normalized using GAPDH (n = 8, *p<0.05).

Given that TNFα is known to activate the NF-κB pathway and myocardin has been shown to decrease NF-κB activation [11], we wished to determine if TNFα could down-regulate myocardin expression and activity in cultured VSMCs. Cultured VSMCs are in a de-differentiated state and more proliferative than differentiated VSMCs. As expression of myocardin specifically and concentration-dependently [7] increases the expression of its target genes, therefore the expression levels of its targets are used to indicate the myocardin activity, especially when myocardin is over-expressed. Therefore, in our study, myocardin activity was defined by measuring the expression levels of myocardin-dependent smooth muscle contractile markers, such as TAGLN, CNN1 and ACTA2. To determine the effect of TNFα on myocardin, we serum-starved VSMCs for 24 h to minimize the effect of serum components. The commonly used concentration of TNFα is 10–100 ng/ml, so we treated the cells with 50 ng/ml TNFα and subsequently performed western blot analysis to check the expression of myocardin and myocardin-dependent downstream contractile marker proteins. We also performed RT-qPCR analysis to examine the effect of TNFα on mRNA levels of myocardin and its target genes, such as *TAGLN*, *CNN1* and *ACTA2*. We found that TNFα down-regulated the expression of myocardin which shows a ∼100 kD band in western blots, and its target genes at both protein and mRNA levels (Fig. 1A,1B). We also examined the effect of TNFα at different concentrations and found that TNFα at 25 ng/ml or higher caused down-regulation of myocardin mRNA and protein expression, and subsequently its downstream target protein ACTA2 (Fig. 1C,1D). Therefore, we use TNFα concentration of 50 ng/ml for all subsequent experiments. Treatment with TNFα for longer time periods (48 or 72 h) yielded similar results to those for 24 h (data not shown). These results indicate that TNFα down-regulates myocardin expression and its activity in cultured VSMCs.

### TNFα down-regulates myocardin expression through the NF-κB pathway

**Figure 2:**
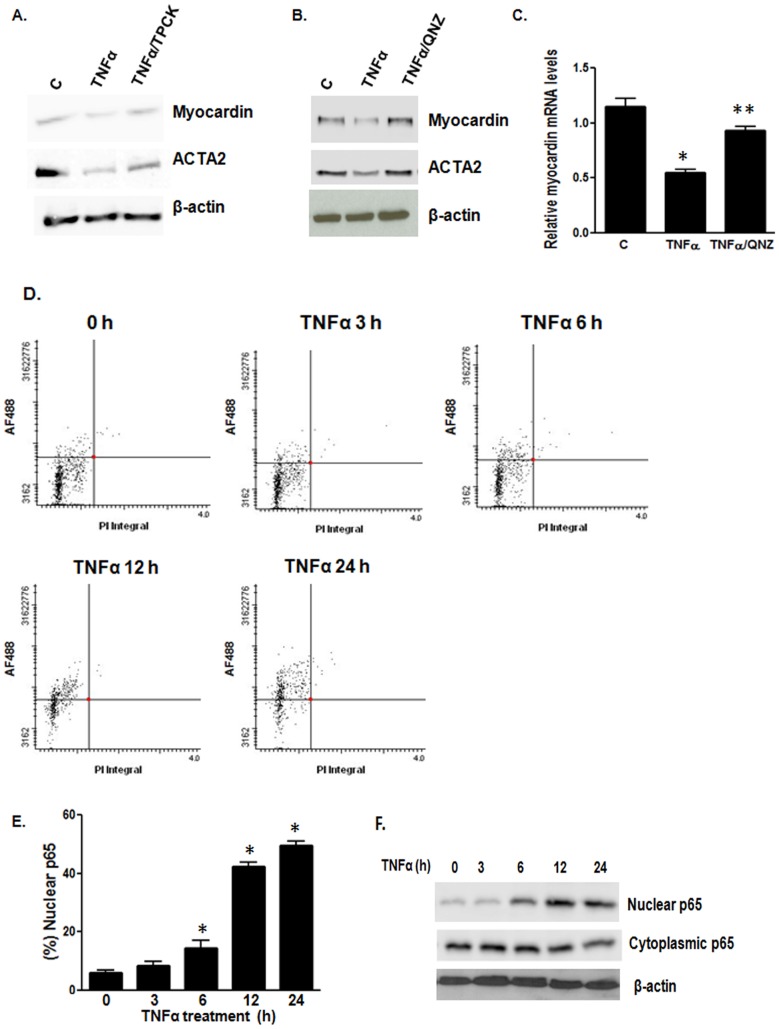
Inhibition of NF-κB blocks TNFα-induced inhibition of myocardin expression and activity. VSMCs were serum-starved for 24 h and treated with TNFα (50 ng/ml). (**A**) Western blot analysis showing the effect of NF-κB inhibitor TPCK (1 µM) on TNFα-induced inhibition of myocardin and contractile marker expression. (**B,C**) Western blot and RT-qPCR analyses showing the effects of NF-κB inhibitor QNZ (1 µM) on TNFα-induced inhibition of myocardin expression and activity compared with the control (C). (**D,E**) VSMCs were treated with TNFα for different time points and the p65 subunit was stained using anti-p65 antibody and AF488-conjugated secondary antibody. The nuclear localization of the p65 subunit was determined using LSC based on AF488 florescence and propidium iodide (PI) nuclear staining. (**F**) VSMCs were treated with TNFα for different time points and cellular and nuclear proteins were extracted separately. Western blot analysis was performed to show localization of the p65 subunit in the cytoplasm and nuclei of VSMCs. qPCR results were normalized using GAPDH (n = 4, *p<0.05).

Next we determined which pathway(s) is involved in TNFα-induced down-regulation of myocardin. TNFα is known to activate the NF-κB and p44/42 MAPK pathways in VSMCs to regulate a variety of cellular functions. Moreover, p44/42 MAPK was shown to phosphorylate myocardin which attenuated the transcriptional activity of myocardin [19]. We co-treated cells with TNFα and NF-κB inhibitor tosyl phenylalanyl chloromethyl ketone (TPCK) or p44/42 MAPK inhibitor PD98059 for 24 h, followed by western blot analysis. Our results revealed that blocking the NF-κB pathway significantly reduced the effect of TNFα on myocardin (Fig. 2A). However, treatment with PD98059 did not significantly affect TNFα effects (data not shown).

As TPCK is an irreversible inhibitor of chymotrypsin, we used another NF-κB inhibitor QNZ (EVP4593), a quinazoline derivative [20], [21]. We co-treated cells with TNFα and NF-κB inhibitor QNZ for 24 h, followed by western blot and RT-qPCR analyses. Our results revealed that blocking the NF-κB pathway with QNZ also significantly reduced the effect of TNFα on myocardin (Fig. 2B, 2C). To confirm that TNFα activates the NF-κB pathway, we used LSC to determine the nuclear translocation of the p65 subunit of NF-κB. In an inactivated state, the NF-κB subunits are localized in the cytoplasm and bound to IκB. Upon receiving an activation signal, IκB is degraded and thep65 subunit translocates to the nucleus, thereby activating several signaling pathways [22]. Nuclear translocation of the p65 subunit is used as a marker of NF-κB activation. We stained the cells using a goat p65 primary antibody, followed by AF488-labelled anti-goat secondary antibody. The nuclear localization of p65 was determined using LSC with the nuclei counterstained with PI. Our results showed that TNFα caused a time-dependent translocation of the p65 subunit into the nuclei (Fig. 2D, 2E), thereby indicating that TNFα causes activation of the NF-κB pathway. Subsequently, western blot analysis with nuclear and cytoplasmic protein fractions also showed an increase in nuclear p65 upon treatment with TNFα (Fig. 2F). These results indicate that TNFα induces activation of the NF-κB pathway, and inhibition of the NF-κB pathway prevents the down-regulation of myocardin by TNFα.

### TNFα decreases contractility and increases proliferation in VSMCs through the NF-κB pathway

**Figure 3:**
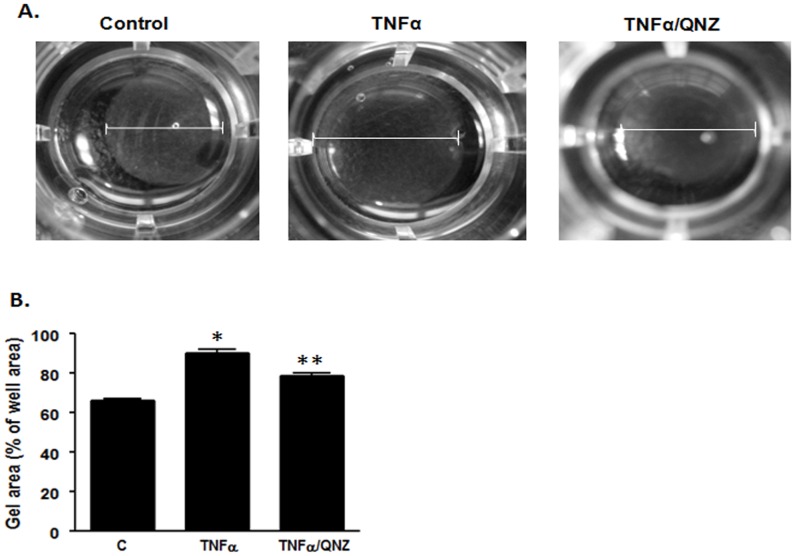
TNFα decreases contractility of VSMCs. VSMCs were serum-starved for 24 h, subcultured into collagen gel matrix and treated with TNFα with or without QNZ for 24 h. (**A**) Representative images of collagen gels showing the change in area of the gel. (**B**) The cross-sectional area was analyzed using ImageJ and plotted on a graph to show the changes in the collagen area (n = 3, *p<0.05).

Myocardin is known to regulate VSMC contractility by increasing the expression of contractile proteins [9]. Hence, down-regulation of myocardin expression should cause a decrease in contractile protein expression and subsequently the contractility of VSMCs. Consistent with this hypothesis, our results using a collagen gel lattice contraction assay showed that TNFα caused a decrease in VSMC contractility, as indicated by the increase in collagen gel lattice area upon TNFα treatment (Fig. 3A, 3B). Moreover, we also found that when the NF-κB pathway was blocked by QNZ, TNFα failed to decrease the contractility of VSMCs, because the area of the collagen gel lattice was significantly smaller than those treated with TNFα alone (Fig. 3A, 3B). These results showed that down-regulation of myocardin by TNFα through the NF-κB pathway results in a decrease in VSMC contractility.

**Figure 4:**
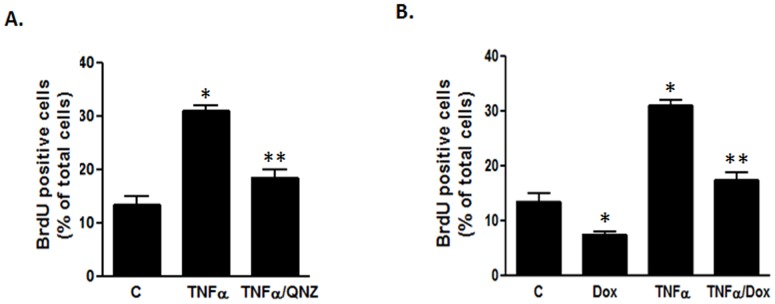
TNFα increases proliferation of VSMCs. VSMCs were serum-starved for 24 h, treated with the indicated drugs for 24 h and labeled with BrdU for 60 min. Cells were then fixed with 80% ethanol and BrdU incorporation was detected using anti-BrdU antibody and AF488-conjugated secondary antibody using LSC. (**A**) Cells were treated with TNFα with and without QNZ. Graph shows the percentage of BrdU positive cells of the total scanned cells. (**B**) Cells were treated with TNFα and/or Dox to induce myocardin expression. Graph shows the percentage of BrdU positive cells of the total number of cells (n = 6, *p<0.05).

We previously showed that myocardin inhibits VSMC proliferation by blocking the NF-κB pathway [11]. Here, we have shown that TNFα activated the NF-κB pathway to down-regulate myocardin. Therefore, we hypothesized that TNFα may stimulate proliferation of VSMCs through this signaling mechanism. We used LSC to perform BrdU incorporation assay to determine the effects of TNFα on DNA synthesis in VSMCs. Our results showed that TNFα caused a significant increase in BrdU incorporation in VSMCs (Fig. 4A), indicating an increase in VSMC proliferation. In addition, blocking the NF-κB pathway with QNZ prevented the TNFα-induced increase in BrdU incorporation (Fig. 4A), supporting the role of NF-κB activation in increased proliferation of VSMCs by TNFα.

Using the T-REx system to over-express myocardin in VSMCs by the addition of Dox, we also found that over-expression of myocardin decreased the BrdU incorporation in VSMCs compared with those in control cells without myocardin over-expression (Fig. 4B). In addition, over-expression of myocardin using Dox significantly attenuated BrdU incorporation induced by TNFα treatment (Fig. 4B). These results have further shown that down-regulation of myocardin by TNFα leads to an increase in VSMC proliferation, which is inhibited by over-expression of myocardin, suggesting a role of myocardin in the regulation of VSMC proliferation.

### TNFα stabilizes myocardin mRNA in differentiated VSMCs

**Figure 5:**
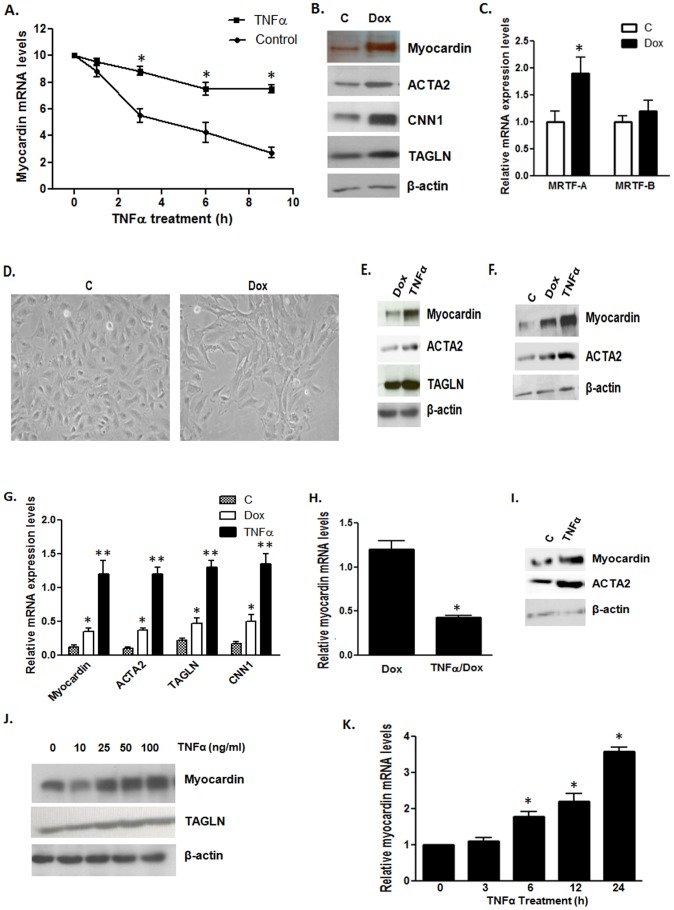
TNFα stabilizes myocardin mRNA which upregulates myocardin expression and activity in differentiated VSMCs. (**A**) VSMCs were serum-starved for 24 h and transcription was blocked using α-amanitin (50 µM). Cells were then treated with TNFα (50 ng/ml) for 1, 3, 6 and 9 h. Graph shows the effect of TNFα on myocardin mRNA degradation. (**B**) VSMCs containing the T-REx system for myocardin over-expression were treated with Dox. Western blot analysis shows the effect of Dox on myocardin and contractile protein expression levels. (**C**) RT-qPCR analysis showing the effect of myocardin over-expression on MRTF-A and B. (**D**) Light microscope images showing the effect of Dox on VSMC morphologies. (**E,F,G**) VSMCs were serum-starved for 24 h and treated with TNFα (50 ng/ml) and Dox for 24 h. Representative western blots and RT-qPCR analysis show the effect of TNFα on myocardin and contractile marker protein and mRNA expression levels compared with those control without Dox (C). (**H**) VSMCs without the T-REx system to over-express myocardin were treated with TNFα and Dox to determine non-specific effects of Dox on myocardin. RT-qPCR analysis shows the effect of TNFα and Dox on myocardin mRNA expression levels compared with the control (C). (**I**) Primary rat aortic VSMCs (passage 2) were serum-starved for 24 h and treated with TNFα (50 ng/ml) for 24 h. Representative western blots show the effect of TNFα on myocardin and contractile marker protein expression levels. (**J,K**) Western blot and RT-qPCR analyses showing the concentration- and time-dependent effects of TNFα on myocardin and contractile marker expression in VSMCs treated with Dox. qPCR results were normalized using GAPDH (n = 7, *p<0.05).

Regulation of mRNA stability is emerging as a key regulator of gene expression [23]. TNFα, in combination with IL-4 or IL-13, can increase vascular cell adhesion molecule-1 (VCAM-1) levels by increasing the mRNA stability of VCAM-1 in synoviocytes [24]. In addition, long-term treatment with TNFα can destabilize cyclooxygenase-2 (COX-2) mRNA in macrophages [25]. Therefore, we determined if TNFα regulates myocardin expression and activity by regulating myocardin mRNA stability. To do so, we serum-starved cells for 24 h and then treated them with α-amanitin to block the transcription. Cells were then treated with TNFα (50 ng/ml) for different time periods (0, 1, 3, 6 and 9 h), followed by RT-qPCR analysis to evaluate the effect of TNFα on myocardin mRNA levels. Interestingly, we found that TNFα prevented the degradation of myocardin mRNA (Fig. 5A).

VSMCs can undergo phenotypic conversion, implicating that they can switch from a differentiated contractile phenotype to a de-differentiated proliferative phenotype, and vice versa [6]. Myocardin controls this phenotypic switching [8]. It is conceivable that different phenotypes of VSMCs possess distinct regulatory mechanisms. We previously showed that over-expression of myocardin using the T-REx system leads to increased differentiation in cultured VSMCs, as indicated by the appearance of more spindle-shaped morphology and increased VSMC contractility [26]. To further confirm TNFα regulation of myocardin mRNA stability in VSMCs, we took advantage of VSMCs harboring the T-REx inducible system to specifically over-express myocardin. Over-expression of myocardin is well known to induce differentiation of VSMCs, resulting in lower proliferation rates and higher contractility [11]. Addition of Dox resulted in increased expression of myocardin and related contractile markers, and more spindle-shaped morphologies (Fig. 5B, 5D). Using RT-qPCR analysis, we observed that over-expression of myocardin results in slight over-expression of MRTF-A, but has no significant effect on MRTF-B (Fig. 5C). We then treated the cells with Dox and subsequently TNFα for 24 h, followed by western blot analysis to check the expression levels of myocardin and related contractile markers. We found that treatment with TNFα further up-regulated the expression levels of myocardin and contractile proteins in VSMCs over-expressing myocardin (Fig. 5E, 5F). As the over-expressed myocardin is mouse in origin, we used Mm_Myocd primers (Qiagen) to detect the over-expressed myocardin using RT-qPCR. We found that similar to the western blot findings, treatment with TNFα further up-regulated the expression levels of myocardin and contractile marker proteins in VSMCs over-expressing myocardin (Fig. 5G). To confirm that the effect of TNFα on myocardin was not due to non-specific effects of Dox, we added Dox together with TNFα in VSMCs expressing only tetracycline repressor (TR) protein, followed by RT-qPCR analysis using human Hs_Myocd primers to detect endogenous myocardin. We found that TNFα still caused a decrease in myocardin expression, but Dox did not have any effect (Fig. 5H). This was consistent with above finding that TNFα down-regulated endogenous myocardin in regularly-cultured de-differentiated VSMCs. In primarily cultured rat aortic SMCs (passage 2), serum-starvation for 24 h was followed by treatment with TNFα for 24 h, and subsequent western blot analysis to determine the expression levels of myocardin and related contractile markers. We found that TNFα increased the expression levels of myocardin and contractile protein (Fig. 5I). Consistently, TNFα also caused both concentration-and time-dependent increase in myocardin expression and activity in differentiated VSMCs (Fig. 5J, 5K). Given that myocardin is constitutively expressed through the T-REx system in the presence of Dox, these results suggest that TNFα may increase myocardin mRNA stability, leading to up-regulation of myocardin expression and activity in more differentiated VSMCs.

### TNFα stabilizes myocardin mRNA through the p44/42 MAPK and NF-κB pathways

**Figure 6:**
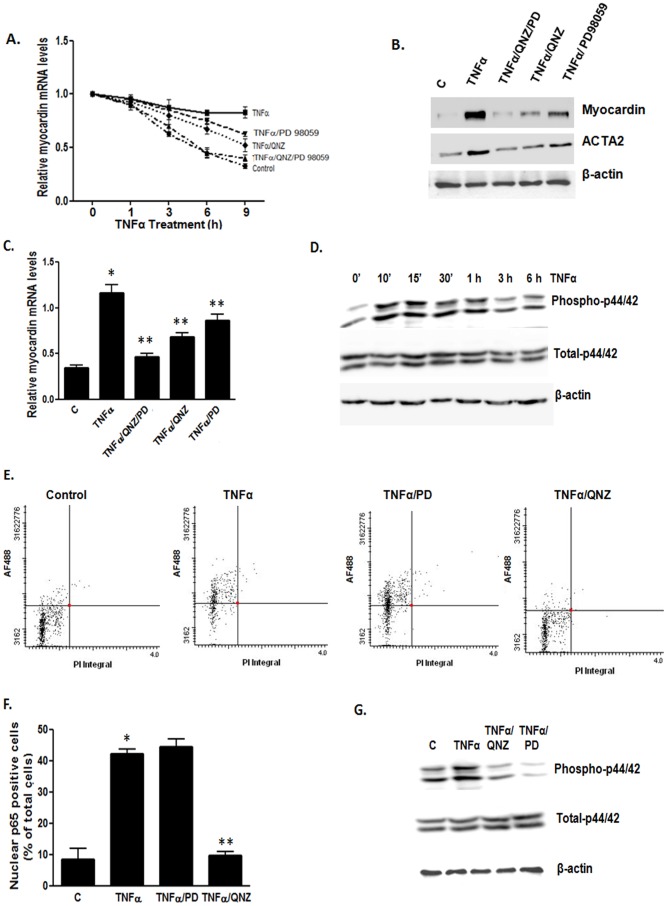
TNFα stabilizes myocardin mRNA through MEK/MAPK and NF-κB pathways. (**A**) VSMCs were serum-starved for 24 h and transcription was blocked using α-amanitin (50 µM). Cells were the treated with TNFα (50 ng/ml) and/or QNZ and PD98059 for 1, 3, 6 and 9 h. Graph shows the effects of QNZ and PD98059 on TNFα-induced decrease in myocardin mRNA degradation. (**B,C**) VSMCs were serum-starved for 24 h and treated with TNFα (50 ng/ml) and/or QNZ and PD98059 for 24 h. All the cells were also treated subsequently with Dox for 24 h. Representative western blots and RT-qPCR analysis show the effect of QNZ and PD98059 on TNFα-induced increase in myocardin and contractile marker protein and mRNA expression levels compared with the control (C) in differentiated cells. (**D**) Western blot analysis showing the activation of the p44/42MAPK pathway by TNFα by observing increase in phosphorylation of p44/42 using phospho-specific antibodies. (**E,F**) VSMCs were treated with TNFα with PD98059 for different time points, p65 subunit was stained using anti-p65 antibody and subsequent AF488-tagged secondary antibody specific to p65 primary antibody. The nuclear localization of the p65 subunit was determined using LSC according to AF488 florescence and propidium iodide (PI) nuclear staining. (**G**) VSMCs were treated with Dox, QNZ and PD98059 for 12 h and then treated with TNFα for 10 min. Western blot analysis shows the activation of p44/42MAPK pathway by TNFα by observing increase in phosphorylation of p44/42 using specific antibodies. qPCR results were normalized using GAPDH (n = 3, *p<0.05).

To elucidate the mechanism for TNFα-dependent increase in myocardin mRNA stability, we co-treated the cells with both TNFα and p44/42 MAPK inhibitor PD98059 or NF-κB inhibitor QNZ or both inhibitors in the presence of inhibition of transcription with α-amanitin. Activation of p44/42 MAPK has been shown to increase TNFα mRNA stability [27]. We observed that both QNZ and PD98059 inhibited the effect of TNFα on myocardin mRNA stability, but the inhibitory effect of QNZ was more significant than that of PD98059 (Fig. 6A). The combined effects of PD98059 and QNZ were even more significant (Fig. 6A), suggesting that both the NF-κB and p44/42 MAPK pathways could play a role in the TNFα-induced increase in myocardin mRNA stability. Consistent with this finding, western blot and RT-qPCR results showed that addition of PD98059 and QNZ inhibited the TNFα-induced increase in myocardin protein expression and its transcriptional activity in VSMCs over-expressing myocardin, with the effect of QNZ being more significant than that of PD98059 (Fig. 6B, 6C). It is interesting to note that the combined effects of PD98059 and QNZ were much more significant than treatment with PD98059 or QNZ alone (Fig. 6B, 6C), implying the involvement of both the pathways. TNFα was also shown to activate the p44/42 MAPK pathway as indicated by an increase in phosphorylation levels of p44/42 MAPK in response to TNFα treatment (Fig. 6D).

Next, we determined if the NF-κB and p44/42 MAPK pathways were parallel, independent or sequential. To do so, we treated the cells with TNFα together with PD98059 or QNZ, followed by examining NF-κB activation using LSC as described above. We found that treatment with PD98059 did not have any effect on TNFα-induced NF-κB activation (Fig. 6E, 6F), and treatment with NF-κB inhibitor QNZ blocked the activation of NF-κB induced by TNFα (Fig. 6E, 6F). Our western blot results showed that inhibition of the NF-κB pathway by QNZ significantly inhibited p44/42 MAPK activation induced by TNFα (Fig. 6G), but p44/42 MAPK inhibitor PD98059 successfully blocked TNFα-induced activation of p44/42 MAPK (Fig. 6G). These findings suggest that NF-κB activation stimulates, at least partially, downstream p44/42 MAPK. Since the combined effects of QNZ and PD98059 were more significant than the effect of QNZ alone, it is also possible that the p44/42 MAPK pathway plays an independent role in regulating myocardin expression.

### TNFα increases contractility of VSMCs over-expressing myocardin

**Figure 7:**
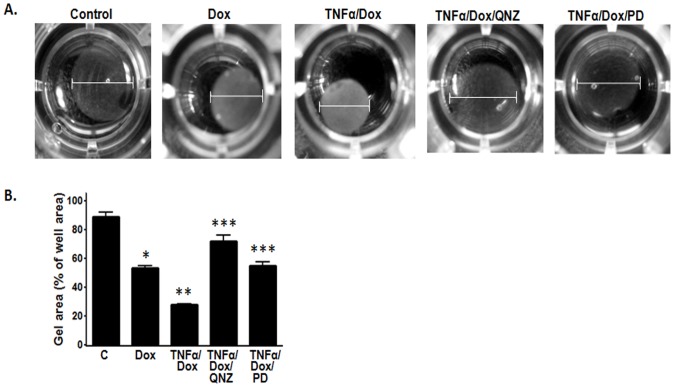
TNFα causes an increase in contractility of myocardin over-expressing VSMCs. VSMCs were serum-starved for 24 h, subcultured into collagen gel matrix and treated with TNFα, Dox, QNZ or PD98059 for 24 h. (**A**) Representative images of collagen gel showing the change in area of the gel. (**B**) The cross-sectional area was analyzed using ImageJ and plotted on a graph to show the change in the area of the collagen (n = 3, *p<0.05).

SMCs with increased expression of contractile marker proteins like CNN1 and TAGLN appear more spindle-shaped and elongated [28]. As myocardin is the master regulator of smooth muscle differentiation and stimulates the expression of downstream contractile marker proteins, SMCs with higher expression of myocardin would appear more contractile and vice versa. Therefore, we determined if TNFα had any effect on the contractility of VSMCs resulting from its effect on myocardin expression. We treated VSMCs harboring the T-REx inducible system with Dox and TNFα for 24 h, followed by performing a collagen gel lattice contraction assay as described above. We found that over-expression of myocardin by addition of Dox caused a reduction in the gel lattice area, indicating an increase in contraction (Fig. 7A, 7B). The addition of TNFα together with Dox caused a further reduction in the gel lattice area (Fig. 7A, 7B), suggesting that TNFα further increased myocardin expression and activity in VSMCs over-expressing myocardin. We also found that blocking the NF-κB or p44/42 MAPK pathway using QNZ or PD98059, respectively, inhibited the effects of TNFα (Fig. 7A, 7B), suggesting the involvement of these pathways in regulation of VSMC contraction.

## Discussion

In the present study, we report that TNFα at the concentration of 25 ng/ml or higher down-regulated endogenous myocardin expression and activity in cultured VSMCs. Commonly used TNFα concentrations with physiological effects are between 10–100 ng/ml [29], [30], [31]. We used concentration of 50 ng/ml for our studies. We also showed that TNFα activated the NF-κB pathway, which was involved in the regulation of myocardin by TNFα. p44/42 MAPK was shown to decrease transcriptional activity of myocardin [19], however we did not observe the involvement of p44/42 MAPK in down-regulation of myocardin by TNFα. TNFα also decreased the contractility of VSMCs, which was reversed by blocking the NF-κB pathway. This effect is explained by our finding that TNFα down-regulated myocardin expression and activity. Myocardin is the master regulator which regulates contractile gene expression in VSMCs [8]. Therefore, a down-regulation of myocardin is expected to lead to a reduction in VSMC contractility as observed in the current study.

Since TNFα activates the NF-κB pathway, and myocardin prevents VSMC proliferation by inhibiting the NF-κB pathway [11], inhibition of myocardin by TNFα through the NF-κB pathway is expected to increase VSMC proliferation. Consistent with this hypothesis, our results have shown that TNFα treatment increased the number of BrdU positive VSMCs, and that inhibiting the NF-κB pathway by QNZ abrogated this effect. We also found that over-expression of myocardin inhibited the increase in VSMC proliferation by TNFα. Over-expression of myocardin alone without TNFα treatment also inhibited VSMC proliferation compared to untreated control. These results clearly point out that by activating NF-κB, TNFα down-regulates myocardin expression and activity, which leads to an increase in VSMC proliferation. It was shown before that TNF-α represses myocardin activation of contractile gene expression in cerebral VSMCs [14] and that NF-κB inhibits myocardin by physically interacting with myocardin [11]. It is conceivable that in our studies the TNFα-activated NF-κB may inhibit endogenous myocardin in cultured VSMCs partially through its direct interaction with myocardin. Given that we observed a decrease in myocardin mRNA levels as well, it is possible that some other mechanism(s) is also involved in NF-κB-mediated regulation of myocardin induced by TNFα. These findings are particularly interesting, because TNFα is known to play a critical role in the development of atherosclerosis. During the development of atherosclerosis, VSMCs de-differentiate and undergo proliferation and migration, leading to development of plaques. Therefore, myocardin, as the master regulator of VSMC differentiation [8], could be involved in the development of atherosclerosis.

Surprisingly, we also found that TNFα prevents the degradation of myocardin mRNA, which leads to accumulation of myocardin mRNA in differentiated cells, and causes a subsequent increase in myocardin expression and activity. However, the further increase in myocardin expression and activity induced by TNFα was only observed when VSMCs are already over-expressing myocardin, i.e., are more differentiated. This effect is not observed in de-differentiated cells, likely because TNFα inhibits myocardin gene expression and there is no significant inhibition of myocardin mRNA degradation when VSMCs are in a de-differentiated state. We also found that both the NF-κB and p44/42 MAPK pathways are involved in the regulation of myocardin by TNFα and that NF-κB regulates the activation of p44/42 MAPK, atleast partially. The increase in myocardin expression and activity by TNFα in VSMCs already over-expressing myocardin also leads to a further increase in contractility. Moreover, consistent with our findings, blocking the NF-κB and p44/42 MAPK pathways significantly inhibits the effect of TNFα on VSMC contractility. TNFα has been previously shown to regulate the mRNA degradation of other proteins like COX-2, IL-8 and VCAM-1 [24], [25], [32]. mRNA stability is regulated by various mechanisms and factors, as is evident by the findings that TNFα mRNA is itself known to be stabilized by other proteins like interferon gamma [33]. Moreover, mRNA of other cytokines, like IL-6, is known to be stabilized too, which leads to increased IL-6 levels [34]. There are several proteins like RNase L which controls the timing of skeletal muscle cell differentiation by orchestrating the regulation of MyoD mRNA stability [35]; poly(A)-binding protein (PABP) which protects mRNAs from ribonuclease attack [36] or A/U-rich RNA-binding protein tristetraprolin (TTP), which is a mRNA destabilizing factor which plays a role in the regulated turnover of many transcripts encoding proteins [37], which regulate the mRNA stability of various mRNAs and they could potentially be involved in this stabilization of myocardin mRNA by TNFα.

**Figure 8:**
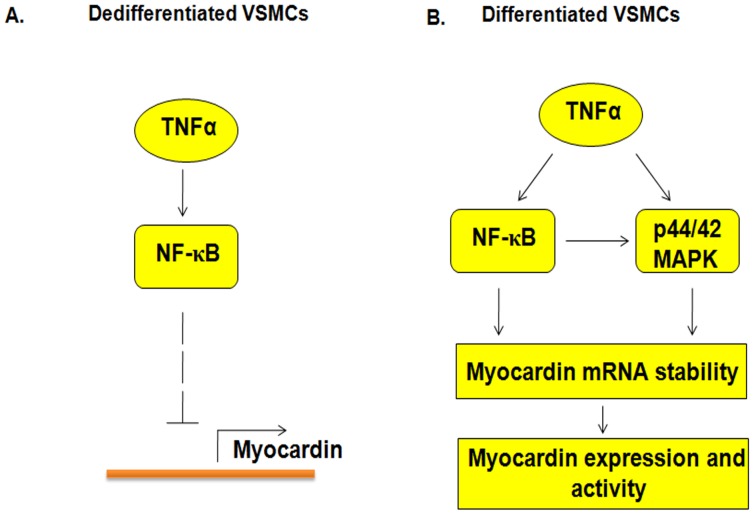
Model of TNFα-mediated myocardin mRNA stability. (**A**) The effect of TNFα in de-differentiated VSMCs. (**B**) The effect of TNFα in differentiated VSMCs.

Interestingly, it was shown that in differentiated macrophages short-term treatment with TNFα increases the stability of COX-2 mRNA, whereas long-term treatment with TNFα destabilizes the COX-2 mRNA, suggesting both pro-inflammatory and protective roles of TNFα during inflammation [25]. In our study, TNFα seems to play different roles in de-differentiated and differentiated VSMCs. In de-differentiated VSMCs, TNFα decreases the expression and activity of myocardin, thereby making them more proliferative and less contractile. However, in differentiated VSMCs, TNFα increases myocardin mRNA stability to further increase the expression and activity of myocardin, thereby making them less proliferative and more contractile. Differential regulation of myocardin by TNFα suggests that TNFα prevents phenotypic switching of VSMCs or prevents de-differentiated cells from becoming differentiated, and vice versa. Recently, it was shown that TNFα decreases myocardin mRNA levels by a kruppel-like transcription factor 4-dependent mechanism in cultured VSMCs [14]. In our study, therefore, TNFα likely regulates myocardin in de-differentiated cells at the transcription level, but in differentiated cells at the post-transcriptional level by increasing myocardin mRNA stability (Fig. 8).

Inflammation is a complex mechanism inside VSMCs. Inflammatory processes have been suggested to repress myocardin and induce an inflammatory SMC phenotype in vivo, which are more proliferative and migratory and lead to development of vascular diseases. We suggest that TNFα plays a role at a later stage during inflammation, when the VSMCs are already dedifferentiated, and causes further dedifferentiation of VSMCs and/or keeps the VSMCs in dedifferentiated state. However, when the VSMCs are differentiated in vivo, TNFα helps keep them in the differentiated state.

Heterogeneity of VSMCs has been previously described and is known to be involved in vascular disease [38], [39]. Epithelioid VSMCs are less differentiated and more proliferative than spindle-shaped VSMCs [38], [39]. We have also shown that there is significantly higher expression of myocardin in spindle-shaped VSMCs compared with epithelioid VSMCs [15]. TNFα might regulate myocardin differently in these two VSMC phenotypes, which in turn could maintain the spindle-shaped and epithelioid phenotypes. However, more work needs to be done to establish this differential regulation and its roles in vascular diseases. This may occur during the development of atherosclerosis. During the initial stages, for example, there is VSMC de-differentiation and down-regulation of contraction proteins, leading to VSMC proliferation and migration. However, during the late stages of atherosclerosis, the plaque is stabilized by formation of a cap containing differentiated VSMCs [40]. Considering that myocardin is the master regulator of VSMC differentiation, we speculate that myocardin could be differentially regulated during the early and late stages of atherosclerosis. However, how VSMCs switch from the de-differentiated phenotype to the differentiated phenotype in the presence of TNFα needs further investigation.

Taken together, our results have revealed that TNFα down-regulates endogenous myocardin expression and activity in cultured VSMCs in the de-differentiated state. Moreover, TNFα also stabilizes myocardin mRNA in differentiated VSMCs resulting from myocardin over-expression, and increases myocardin expression and activity, suggesting differential regulation of myocardin by TNFα depending on the differentiation status of VSMCs. The significance of our study is that TNFα could regulate myocardin and smooth muscle function differentially, depending on the differentiation states of VSMCs. This could be involved in maintenance of different phenotypes of VMSCs and also play a role in the different stages of atherogenesis. Although much work needs to be done to further establish the detailed mechanisms by which myocardin is differentially regulated, our findings have suggested an important role for TNFα in the regulation of myocardin in VSMCs, and likely the pathogenesis of vascular diseases, such as atherosclerosis.
